# Comparison of Protein and mRNA Expression Evolution in Humans and Chimpanzees

**DOI:** 10.1371/journal.pone.0000216

**Published:** 2007-02-14

**Authors:** Ning Fu, Ines Drinnenberg, Janet Kelso, Jia-Rui Wu, Svante Pääbo, Rong Zeng, Philipp Khaitovich

**Affiliations:** 1 Key Lab of Systems Biology, Shanghai Institutes for Biological Sciences, Chinese Academy of Sciences, Shanghai, China; 2 Max-Planck-Institute for Evolutionary Anthropology, Leipzig, Germany; 3 Institute for Computational Biology, Shanghai Institutes for Biological Sciences, Chinese Academy of Sciences, Shanghai, China; Temasek Life Sciences Laboratory, Singapore

## Abstract

Even though mRNA expression levels are commonly used as a proxy for estimating functional differences that occur at the protein level, the relation between mRNA and protein expression is not well established. Further, no study to date has tested whether the evolutionary differences in mRNA expression observed between species reflect those observed in protein expression. Since a large proportion of mRNA expression differences observed between mammalian species appears to have no functional consequences for the phenotype, it is conceivable that many or most mRNA expression differences are not reflected at the protein level. If this is true, then differences in protein expression may largely reflect functional adaptations observed in species phenotypes. In this paper, we present the first direct comparison of mRNA and protein expression differences seen between humans and chimpanzees. We reproducibly find a significant positive correlation between mRNA expression and protein expression differences. This correlation is comparable in magnitude to that found between mRNA and protein expression changes at different developmental stages or in different physiological conditions within one species. Noticeably, this correlation is mainly due to genes with large expression differences between species. Our study opens the door to a new level of understanding of regulatory evolution and poses many new questions that remain to be answered.

## Introduction

Microarray technology has become the method of choice for studying messenger RNA (mRNA) expression levels for thousands of genes in cells or tissues subjected to different conditions, or derived from different species [1]. Biological processes, however, are normally driven by proteins. Still, methods for studying protein expression levels are much more laborious and costly, and more limited in scope, than mRNA measurements. The methodologies based on two-dimensional gel electrophoresis or shotgun mass spectrometry, for instance, are imprecise and biased towards highly expressed proteins [2]. Introduction of quantitative proteomics approaches, such as isotope coded affinity tags (ICAT), allow obtaining more precise protein abundance estimates for a broader range of protein concentrations. However, this approach is currently limited to the identification of a few hundred proteins in a given sample [3]–[5].

For these reasons most current studies use mRNA expression levels as a proxy for estimating functional differences that occur at the protein level. This strategy assumes that differences in mRNA levels actually reflect differences in protein expression. Most studies addressing this issue to date, however, describe the correlation between mRNA and protein expression as moderately or weakly positive with correlation coefficients ranging from 0.2 to 0.6 [4]–[7]. This lack of agreement can in part be explained by the technical imprecision of methods used to determine their expression levels [2]. Biologically, the discrepancies between mRNA and protein expression can be caused by posttranscriptional regulation, as well as differences in mRNA and protein turnover rates [2], [7].

Further, all studies to date, investigated mRNA and protein expression correlation within one species in different developmental or physiological states, or among tissues within the same organism [4]–[11]. Unlike changes observed in response to functional stimuli within an organism or a species, a large proportion of mRNA expression differences observed between mammalian species are caused by random genetic mutations and, analogous to the DNA sequence changes, appear to have no functional consequences (see [12] for the review). This may be due to the fact that many or most mRNA expression differences are not reflected at the protein level and thus do not exert any influence on the organisms phenotype. If this is true, we expect to see much weaker correlation between mRNA and protein expression differences observed between species than between the ones observed in response to functional stimuli. Furthermore, the observed protein expression differences would then largely reflect functional adaptations observed in species phenotypes. Such a finding would be of particular importance for studies of human evolution, where, despite intensive effort, the identification of genomic or mRNA expression changes underlying human-specific phenotypic features has been a daunting and hitherto largely elusive task [13], [14]. On the other hand, the finding that evolutionary changes in mRNA expression are reflected at the protein level would have important implications for our views of regulatory evolution.

## Results

To investigate whether mRNA expression differences observed between humans and chimpanzees are reflected in differences in protein expression, we measured mRNA expression levels of all annotated genes in six human and six chimpanzee livers, using microarray probes that perfectly matched the human as well as the chimpanzee genome sequences. In addition, we measured protein expression levels in six independent pools of tissue extracts, each containing two of the human or two of the chimpanzee samples, respectively, in two sets of experimental replicates, using ICAT profiling (Materials and Methods).

**Figure 1:**
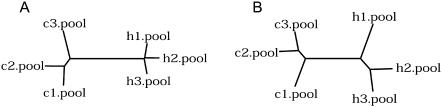
Schematic representation of gene expression variation within and between species on mRNA (A) and protein (B) levels. The trees are inferred from the mean of the squared difference of expression intensities for 98 genes detected on both mRNA and protein levels. Each pool represents an average expression in two chimpanzee (c) or two human (h) individuals. Additionally, protein measurements are based on two independent experimental replicates.

First, we tested our ability to distinguish the human and chimpanzee samples based on protein expression. Using either tree construction or hierarchical clustering based on 113 proteins detected in all 12 samples, we find that the human and the chimpanzee protein expression profiles are clearly distinct (Figure 1B, Figure S1). Further, although a direct comparison between mRNA and protein expression variation is not possible, the overall extent of intra- and inter-species variation appears to be similar (Figure 1). Compared with mRNA expression, protein expression exhibits greater intra-species variation. Large experimental variation associated with protein expression measures is likely, at least in part, to explain this variation (Materials and Methods).

**Figure 2:**
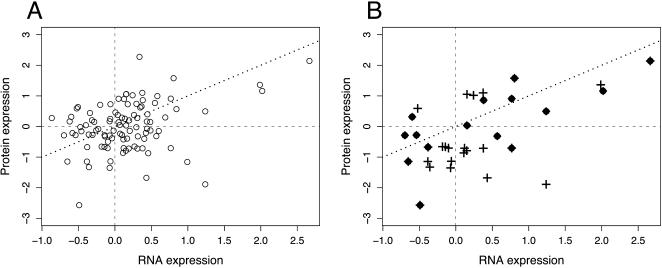
Comparison of protein and mRNA expression differences between humans and chimpanzees. Comparisons are shown for 98 genes, detected on both mRNA and protein levels (A), and for 33 genes showing significant differences in mRNA (⧫) or protein (+) expression between the species (B). Six genes significantly different on both mRNA and protein expression levels are shown using overlapping labels. The names and the functional annotation of the 33 genes are included in table S5. Expression differences are shown using a base-two logarithmic scale. The dotted line represents an ideal regression line (α = 0, β = 1).

Next, we tested whether gene expression differences between humans and chimpanzees observed on the mRNA level reflect differences in protein expression. For 98 genes detected on both protein and mRNA levels, we find a significant positive correlation between differences in mRNA and protein expression (*R* = 0.33, *p*<0.001) (Figure 2A). However, the vast majority of the 98 genes do not show large expression differences between the species, neither in terms of protein levels nor in terms of mRNA levels. This is not surprising, given that approximately 90% of genes do not differ significantly in their mRNA expression levels in somatic tissues between humans and chimpanzees [14]. When we restrict our analysis to 15 genes with a significant mRNA expression difference between species (Student's t-test, *p*<0.01, FDR = 4%), we find a much stronger correlation (*R* = 0.72, *p* = 0.002). Further, we find a similar correlation (*R* = 0.55, *p* = 0.005) on the protein level (Student's t-test, *p*<0.01, FDR = 3%) for 24 genes differently expressed between humans and chimpanzees (Figure 2B).

Finally, we tested whether the observed correlation can be reproduced using the protein expression data from each of the two sets of experimental replicates separately. We find a significant correlation between mRNA and protein expression differences between humans and chimpanzees for 143 and 159 genes detected in the first and second sets of experimental replicates (*R* = 0.37, *p*<0.000005 and *R* = 0.28, *p*<0.0005, respectively) as well as for 24 and 23 genes with a significant mRNA expression difference between species (Student's t-test, *p*<0.01, FDR = 5%) (*R* = 0.68, *p*<0.0005 and *R* = 0.48, *p*<0.05, respectively) (Figure S2).

## Discussion

We reproducibly find a significant positive correlation between mRNA expression and protein expression differences seen between humans and chimpanzees. We find this positive correlation not only for genes showing significant expression differences between species, but for all detected genes. Further, it is present in each set of replicates as well as the combined dataset. Furthermore, the observed correlation is not simply the result of a strong effect in a small subset of genes, as indicated by bootstrapping all detected genes 10,000 times (*p* = 0.01 and *p* = 0.05 for the first and the second set of replicates, respectively) (Figure S3).

Although our observation is currently limited to a relatively small number of samples from a single tissue, we are able to reproduce the result in two independent sets of experimental replicates, despite the substantial experimental noise associated with protein expression measurements. In fact, since technical variation is expected to be random, the actual correlation is likely to be better than that observed. Further, the observed correlation exists even though we use largely non-overlapping sets of individuals for the mRNA and protein expression measurements. The inclusion of variation caused by expression differences between samples used for protein and mRNA measurements means that we expect the actual correlation between protein and mRNA expression to be better than that observed. Together, these features indicate that the correlation we observe, though weakened by technical effects, is unlikely to be spurious.

Genes detected on the protein level in this study tend to have a higher average mRNA expression level than other genes. However, their distribution covers the entire expression spectrum (Figure S4). With current data it is not possible to say to what extent the observed results may apply to a broader set of genes or to other tissues.

We find that for the 98 genes detected on both mRNA and protein levels in the two datasets, there is a significant enrichment for genes involved in metabolic processes (GO ontology reference) when compared to genes expressed on the mRNA level alone (Table S1). However, of these the 33 genes differently expressed between the species and showing the strongest correlation do not differ significantly in their function from the bulk of detected genes. Thus, the protein and mRNA expression differences that correlate the most do not appear to cluster in any particular biological process.

When all detected genes are considered, the strength of the correlation between expression differences seen on the mRNA and protein levels appears weak (*R* = 0.28–0.37). However, similar correlations have been reported in comparisons between different developmental stages in mice (*R* = 0.18) [7], between yeasts cultured on rich or minimal media (*R* = 0.45) [6], or between two murine hematopoetic cell lines (*R* = 0.59) [5], where the results for all detected genes were reported. Since some proteins are detected in just one of the two datasets, our total analysis is based on comparison of protein and mRNA expression levels for 206 genes. Other studies have used similar numbers of proteins for comparison of ICAT and mRNA microarray methodologies (N = 245, 289, 425) [3]–[5]. Thus, the correlation we find between evolutionary differences in protein and mRNA expression is comparable, or at least not worse than, those observed between different physiological states in an organism.

Further, approximately 90% of genes expressed in liver do not differ significantly in their mRNA expression levels between humans and chimpanzees. Thus, for the majority of these we cannot expect to find any correlation between proteins and mRNA based on the expression differences between species. As expected, when we limit our analysis to genes that show significant differences between species, the correlation improves considerably (*R* = 0.5–0.7, see Results). Still, this result is based on several dozens of genes and needs to be further confirmed using a much larger dataset. Nonetheless, these results provide the first indication that the mRNA expression differences detected between humans and chimpanzees may to a large extent reflect protein expression differences.

This indication, if confirmed, poses new questions, rather than resolving existing ones. Namely, if the vast majority of evolutionary differences in mRNA expression between closely related species does not affect phenotype, then the majority of evolutionary differences in protein expression are also likely to be of little or no consequence for the phenotype. Alternatively, the observed correlation may indicate that a considerable number of mRNA expression differences between species, particularly those of large amplitudes, might have some functional effect. Nonetheless, before any general conclusions regarding the relation of mRNA and protein expression differences between species can be made, further studies covering more genes and tissues are necessary.

## Materials and Methods

### Tissue samples and microarray data collection

All chimpanzee individuals used in this study belonged to the eastern chimpanzee population. All tissue samples were obtained from individuals that suffered sudden deaths for reasons other than their participation in this study and without any relation to the tissues used. All samples had no histological abnormalities and showed no detectable RNA degradation, indicating good tissue preservation (Table S2). Tissue samples from four of the six chimpanzee individuals were used in both protein and mRNA expression analysis, while the remaining tissue samples used for protein and mRNA expression analysis did not overlap. Human and chimpanzee samples were matched with respect to sex and relative age as closely as possible, with no sex or age bias shared between the samples used for mRNA and protein measurements (Table S2).

mRNA expression levels were determined by analyzing a published gene expression dataset for six human and five chimpanzee livers with added expression data taken from one chimpanzee individual [14]. All samples in this dataset were prepared, labeled and hybridized to Affymetrix® HG U133plus2 arrays in one batch, following the procedure described in [14]. Namely, total RNA was extracted from 100 mg of frozen tissue dissected from the peripheral part of liver lobe in the TRIZol® reagent using an electric homogenizer (Schütt) at 3,000 rpm. Further, total RNA was isolated according to the TRIZol® manufacturer's instructions and purified with a QIAGEN® MiniElute® kit, following the protocol supplied by the manufacturer without modifications. All samples were processed, labeled and hybridized to the microarrays following the protocol described in the GeneChip® Expression Analysis Technical Manual (http://www.affymetrix.com/support/technical/manuals.affx) without any modifications.

All samples had a high and comparable RNA quality, as determined by both the ratio of 28S to 18S ribosomal RNAs, estimated using the Agilent® 2100 Bioanalyser® system (Table S2), and the signal ratios between the probes for the 5′ and 3′ ends of the mRNAs of GAPDH, which are used as quality controls on Affymetrix® microarrays. All primary expression data are publicly available at the ArrayExpress database (http://www.ebi.ac.uk/arrayexpress/), accession number E-AFMX-11.

### Microarray data analysis

Prior to microarray data analysis, we removed all oligonucleotide array probes that did not perfectly match both human and chimpanzee genome sequences, or had a significant difference between these two species in their hybridization patterns relative to the other probes of the same set, as described in [14]. This reduced the number of probe sets available for analysis on each array from 54,675 to 51,522. mRNA expression values were calculated using the Robust Multichip Average (rma) algorithm [15]. Detection *p*-values were calculated using the “mas5calls” function included in the Bioconductor “affy” package (http://www.bioconductor.org/). Only probe sets with expression levels detected in at least two samples (detection *p*-value<0.05) were considered for further analysis. This further reduced the number of probe sets available for analysis to 23,856. Since many genes are represented by more than one probe set on the Affymetrix® HG-U133Plus2 arrays, we calculated the mean expression values for each gene, as defined by the RefSeq annotation, Affymetrix® annotation tables, December 2005 version (http://www.affymetrix.com/index.affx).

### Protein data collection

Protein expression levels were determined in liver samples from six humans and six chimpanzees. Prior to protein isolation, we combined samples from two individuals of one species in one pooled sample, thus creating 3 independent sample pools for each species (Table S2). The pooled samples were minced, washed with ice-cold PBS to remove blood, and homogenized in an ice-cold lysis buffer (8 M urea, 4% CHAPS, 65 mM DTT, 40 mM Tris, 200 mg of tissue/1 ml), using an electric homogenizer (Glas-Col). The homogenized solution was sonicated for total of 3 minutes (5 seconds sonication time with 10 seconds intervals). After that, the sample solution was then centrifuged at 10,000 g for 1 hour at 4°C. The protein supernatant was transferred into a new tube and then frozen at −80°C.

ICAT labeling was performed using a Cleavable ICAT^TM^ Reagent Kit (Applied Biosystems, Foster City, CA) according to the manufacturer's guidelines, with the few modifications described in [16]. Orthogonal 2D-LC-MS/MS was performed in an ion-trap mass spectrometer (LTQ, Thermo Finnigan). The system was fitted with a strong cation exchange column (CTIBiphase, SCX 0.32 mm×5 cm, COLUMN Techology Inc.) and two C18 reversed-phase columns (sample trap, C18,300 Å, COLUMN Techology lnc). For the ion exchange step, we used a pH gradient with steps at pH 3.0, 3.5, 4.0, 4.5, 5.0, 5.5, 6.0, 7.0, 8.0, 8.5. For the reverse phase step, we used 0.1% formic acid in either water (buffer A) or ACN (buffer B). We used buffer B gradient from 0 to 35% during the first 165 minutes, and from 35% to 95% in the following 15 minutes. One full MS scan was then followed by ten MS/MS scans on the ten most intense ions from the MS spectrum, according to a dynamic exclusion setting: repeat count, 2; repeat duration, 30 seconds; exclusion duration, 90 seconds. In each measurement, individual samples were measured together with a common reference sample comprised of all 12 samples used in this study. In the second set of replicates, the ICAT labels used for individual samples and common reference were inverted.

### Protein expression analysis

For protein identification and statistical validation, the acquired MS/MS spectra were automatically searched against *ipi.human* version 3.07 database using the Turbo SEQUEST program in the BioWorks™ 3.1 software suite. SEQUEST results were filtered using the following parameters: Xcorr (a cross-correlation value) was greater or equal to 1.9 for the peptides in a +1 charged state, 2.2 for ones in a +2 charged state and 3.75 for ones in a +3 charged state, where the delta Cn is greater or equal to 0.1. The accepted peptide quantification was achieved using the Relex software and partial manual verification. The peptide database search was carried out with the following parameters: peptide mass tolerance = 3.0000; fragment ion tolerance = 0.0000; maximum number of internal cleavage sites = 2; number of allowed errors in matching auto-detected peaks = 1; mass tolerance for matching auto-detected peaks = 1.0000.

Following this procedure, we identified and quantified expression levels for 169 proteins in all 6 sample pools in the first experiment, and 190 proteins in the second experiment. 113 of these proteins were identified in both experiments. Protein expression was calculated as a ratio of the protein expression level in a pooled sample to the protein expression level in a common reference sample comprised of all 12 samples used in this study. For further analysis, the ratios were base-two logarithm transformed.

We found that protein expression measures among individuals of the same species are associated with large variation. Even though we found a significant correlation of protein expression values across most of the pairs of individuals, the average correlation coefficient was relatively low (*R* = 0.31). Further, the levels of variation observed between individuals within a species ranged widely, as reflect by the correlation coefficient distribution (Figure S5). We find that the lowest observed correlation was due to just two points with the greatest influence on the linear model fit indicated by Cook's distances. Removal of points with the Cook's distance greater than 0.1, (less than 5% of the total points) improved the correlation observed among individuals within the species (Figure S5).

More importantly, however, we found that the correlation between replicate measurements of the same protein sample were substantially better than the correlations observed among the individuals (mean *R* = 0.48, range 0.38–0.60, *p*<0.0005) (Figure S5). Given that the experimental replicates were prepared in two completely independent routines, including flipping mass tags and measuring them in two series separated by more than a month, the observed correlations indicate that the protein measurements, albeit noisy, accurately reflect protein abundance levels. Further, we can conclude that experimental variation is unlikely to explain more than a half of the total variation observed among individuals of the same species.

### Combined data analysis

We combined protein and mRNA expression data on a gene-by-gene basis using RefSeq annotation (http://www.ncbi.nlm.nih.gov/RefSeq/). Both mRNA and protein expression measurements were available for 143 genes represented by 682 probe sets on the microarray for the first set of experimental replicates for 159 genes represented by 572 probe sets on the microarray for the second set of experimental replicates, and for 98 genes represented by 442 probe sets on the microarray for both sets. Protein and mRNA expression values for 143 and 159 genes detected in the first and second sets of experimental replicates are listed in tables S3 and S4, respectively. For the correlation analysis, we calculated differences between humans and chimpanzees as a difference between mean expression values within species, for all genes detected on both mRNA and protein levels. Genes differently expressed between humans and chimpanzees either on protein or mRNA expression levels were identified using Student's t-test. The false discovery rate (FDR) was calculated in 1,000 random permutations of sample labels. The positive correlation observed between mRNA and protein expression differences did not depend a great deal on the significance cutoff chosen to define differently expressed genes. Thus, for the 143 proteins detected in the first set of experimental replicates, at cutoff *p*<0.005, FDR = 2%, we found 18 genes with correlation *R* = 0.70, *p* = 0.001; at cutoff *p*<0.01, FDR = 4%, we found 24 genes with correlation *R* = 0.68, *p* = 0.0003; and at cutoff *p*<0.02, FDR = 6%, we found 31 genes with correlation *R* = 0.58, *p* = 0.0006.

For tree construction, the distances between each pair of samples were calculated as a mean of squared differences between the intensities of expressed genes. We used the resulting data matrixes to build UPGMA trees using PHYLIP [17]. For the tree based on mRNA expression, we created pooled samples by calculating the mean expression value for each gene based on expression levels in two individuals of the same species (Table S2). For the tree based on protein expression, we calculated the mean expression value for each gene based on expression levels in two experimental replicates (Table S2). Thus, for both mRNA and protein data, such pooling reduces both individual and experimental variation.

Hierarchical clustering of protein data was completed using average linkage clustering, and visualized using Cluster and TreeView software, respectively (http://rana.lbl.gov/EisenSoftware.htm). Before clustering, protein expression values were normalized on a gene-by-gene basis and the mean expression of each sample was centered to the same value. Gene ontology analysis was carried out using publicly available on-line statistical package (FUNC, http://func.eva.mpg.de/) using a hypergeometric test. Genes were assigned GO annotations using the Ensembl annotation tool BioMart (http://www.ensembl.org/Multi/martview).

## Supporting Information

Figure S1 Hierarchical clustering of 12 liver samples based on protein expression levels of 113 genes, detected in all samples
(0.03 MB DOC)

Figure S2 Comparison of protein and mRNA expression differences between humans and chimpanzees
(0.24 MB DOC)

Figure S3 Bootstrap analysis of Pearson's correlation coefficients and correlation p-values observed in the first and in the second set of experimental replicates
(0.10 MB DOC)

Figure S4 Distributions of mRNA expression levels (on logarithmic two scale) for 143 genes identified on the protein expression level (left), and remaining genes detected on the mRNA expression level
(0.08 MB DOC)

Figure S5 Distribution of Pearson's correlation coefficients for the comparisons between individuals within a species and between the experimental replicates
(0.11 MB DOC)

Table S2 Sample information
(0.02 MB XLS)

Table S3 mRNA and protein expression levels for 143 proteins detected in the first set of experimental replicates
(0.08 MB XLS)

Table S4 mRNA and protein expression levels for 159 proteins detected in the second set of experimental replicates
(0.09 MB XLS)

Table S1 GO categories with significant overrepresentation of 98 genes detected on the protein and mRNA levels
(0.04 MB XLS)

Table S5 Functional annotation of 33 genes with significant expression difference between humans and chimpanzees
(0.03 MB XLS)
