# Supplementary material for: Comparison of Protein and mRNA Expression Evolution in Humans and Chimpanzees
Source: PLoS One. 2007 Feb 14;2(2):e216. doi: 10.1371/journal.pone.0000216 (PMC1789144; doi:10.1371/journal.pone.0000216)
Supplement: Figure S1 — Hierarchical clustering of 12 liver samples based on protein expression levels of 113 genes, detected in all samples (0.03 MB DOC) [file pone.0000216.s001.doc]

**
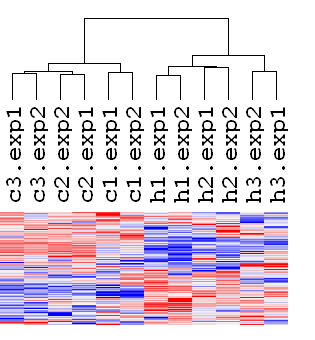
**

**Figure S1** Hierarchical clustering of 12 liver samples based on protein expression levels of 113 genes, detected in all samples.

Each sample is a pool of two chimpanzee (**c**) or two human (**h**) individual samples, measured in the first (**exp1**) or the second (**exp2**) set of experiments. Colors represent the ratio of protein expression in a given sample compared to a common reference, red – higher expression, blue – lower expression, gray – no difference.
