# Supplementary material for: Comparison of Protein and mRNA Expression Evolution in Humans and Chimpanzees
Source: PLoS One. 2007 Feb 14;2(2):e216. doi: 10.1371/journal.pone.0000216 (PMC1789144; doi:10.1371/journal.pone.0000216)
Supplement: Figure S2 — Comparison of protein and mRNA expression differences between humans and chimpanzees (0.24 MB DOC) [file pone.0000216.s002.doc]

**A B**


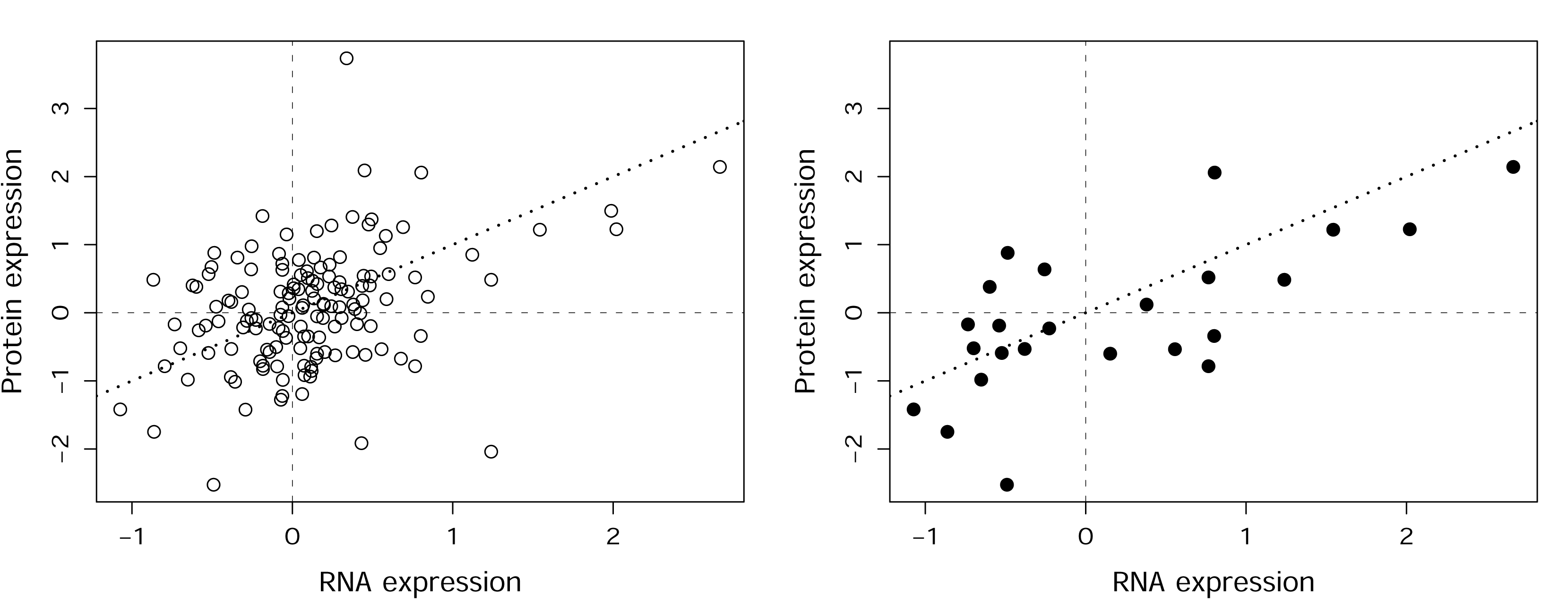


**C D**


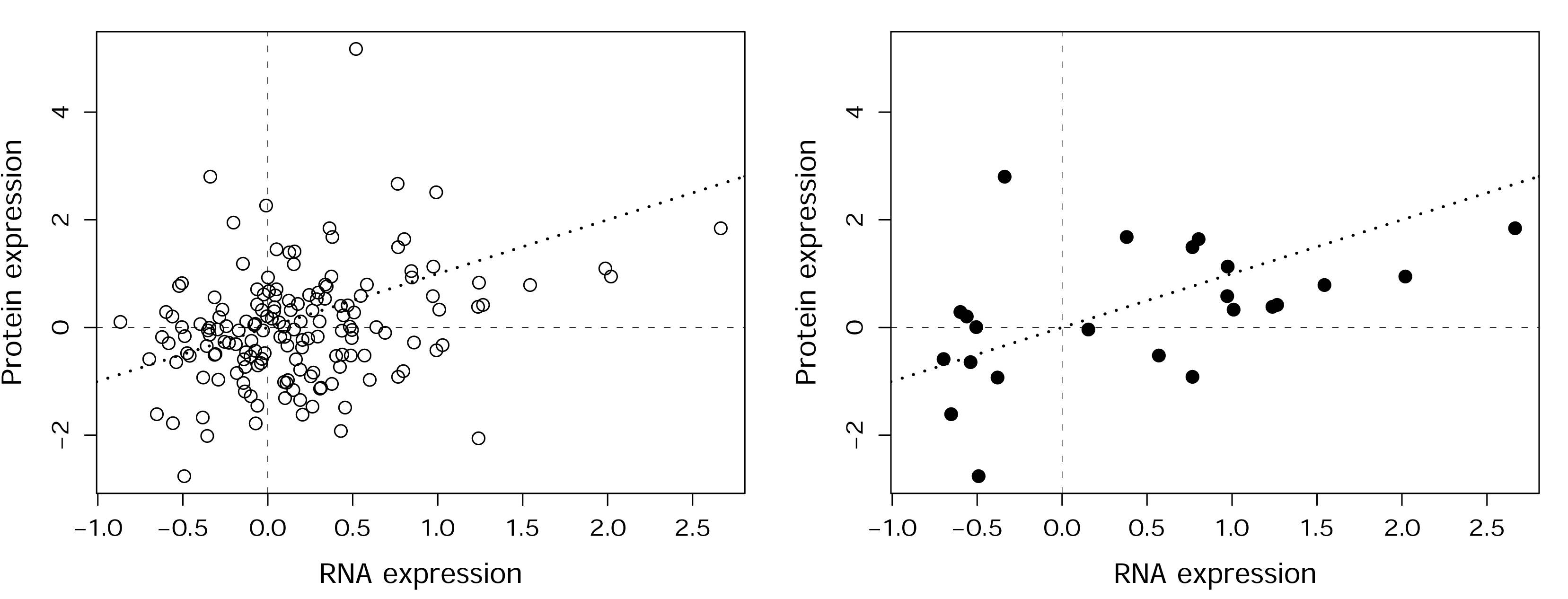


**Figure S2** Comparison of protein and mRNA expression differences between humans and chimpanzees.

Comparisons are shown for 143 and 159 genes detected on both mRNA and protein levels using protein data from the first and the second set of experimental replicates, respectively (**A** and **C**), and for 24 and 23 genes showing significant differences in mRNA expression in these two sets (**B** and **D**). No significant differences in protein expression were detectable in 6 samples. Expression differences are shown using a base-two logarithm scale. The dotted line represents an ideal regression line (*α*=0, *β*=1).
