# Supplementary material for: Comparison of Protein and mRNA Expression Evolution in Humans and Chimpanzees
Source: PLoS One. 2007 Feb 14;2(2):e216. doi: 10.1371/journal.pone.0000216 (PMC1789144; doi:10.1371/journal.pone.0000216)
Supplement: Figure S3 — Bootstrap analysis of Pearson's correlation coefficients and correlation p-values observed in the first and in the second set of experimental replicates (0.10 MB DOC) [file pone.0000216.s003.doc]

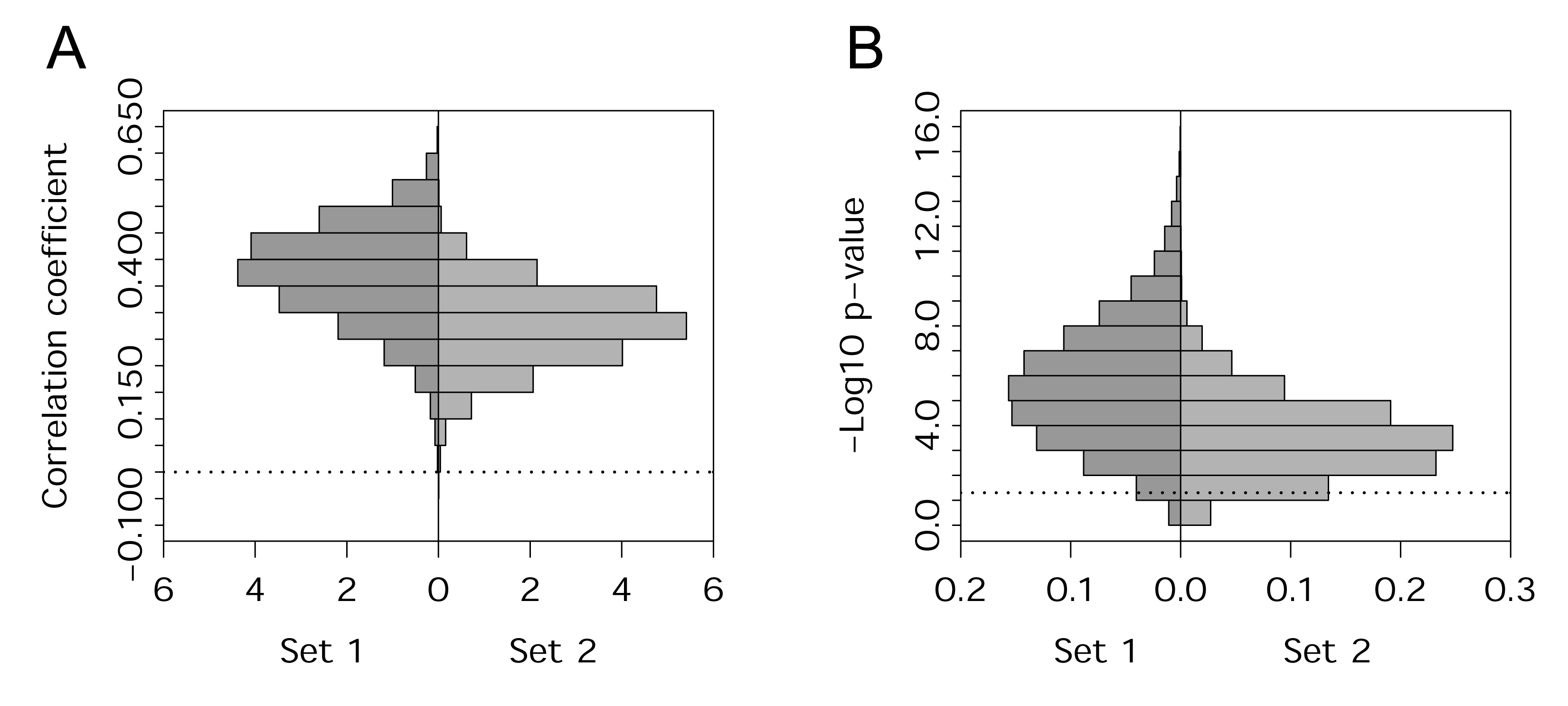


**Figure S3** Bootstrap analysis of Pearson's correlation coefficients and correlation *p*-values observed in the first and in the second set of experimental replicates.

Shown are the distributions of Pearson's correlation coefficients (**A**) and correlation *p*-values (on negative logarithm ten scale) (**B**) measured in 10,000 bootstrap permutations of 143 genes (left, darker shade) and 159 genes (right, lighter shade) detected in the first and in the second set of experimental replicates. The dotted line represents zero correlation value (**A**) or *p*-value threshold=0.05 (**B**).
