# Supplementary material for: Comparison of Protein and mRNA Expression Evolution in Humans and Chimpanzees
Source: PLoS One. 2007 Feb 14;2(2):e216. doi: 10.1371/journal.pone.0000216 (PMC1789144; doi:10.1371/journal.pone.0000216)
Supplement: Figure S4 — Distributions of mRNA expression levels (on logarithmic two scale) for 143 genes identified on the protein expression level (left), and remaining genes detected on the mRNA expression level (0.08 MB DOC) [file pone.0000216.s004.doc]

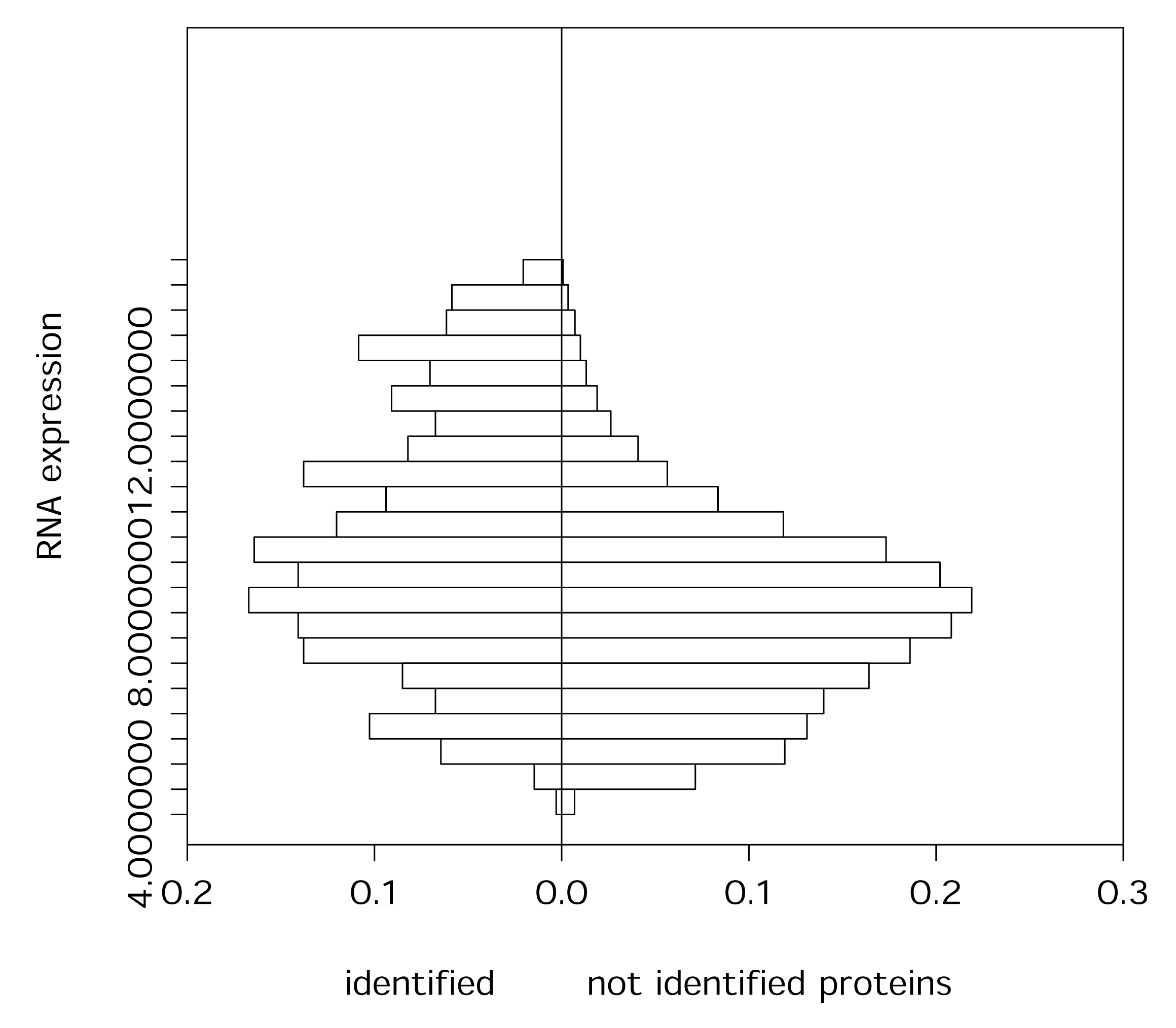


**Figure S4** Distributions of mRNA expression levels (on logarithmic two scale) for 143 genes identified on the protein expression level (left), and remaining genes detected on the mRNA expression level.
