# Supplementary material for: Comparison of Protein and mRNA Expression Evolution in Humans and Chimpanzees
Source: PLoS One. 2007 Feb 14;2(2):e216. doi: 10.1371/journal.pone.0000216 (PMC1789144; doi:10.1371/journal.pone.0000216)
Supplement: Figure S5 — Distribution of Pearson's correlation coefficients for the comparisons between individuals within a species and between the experimental replicates (0.11 MB DOC) [file pone.0000216.s005.doc]

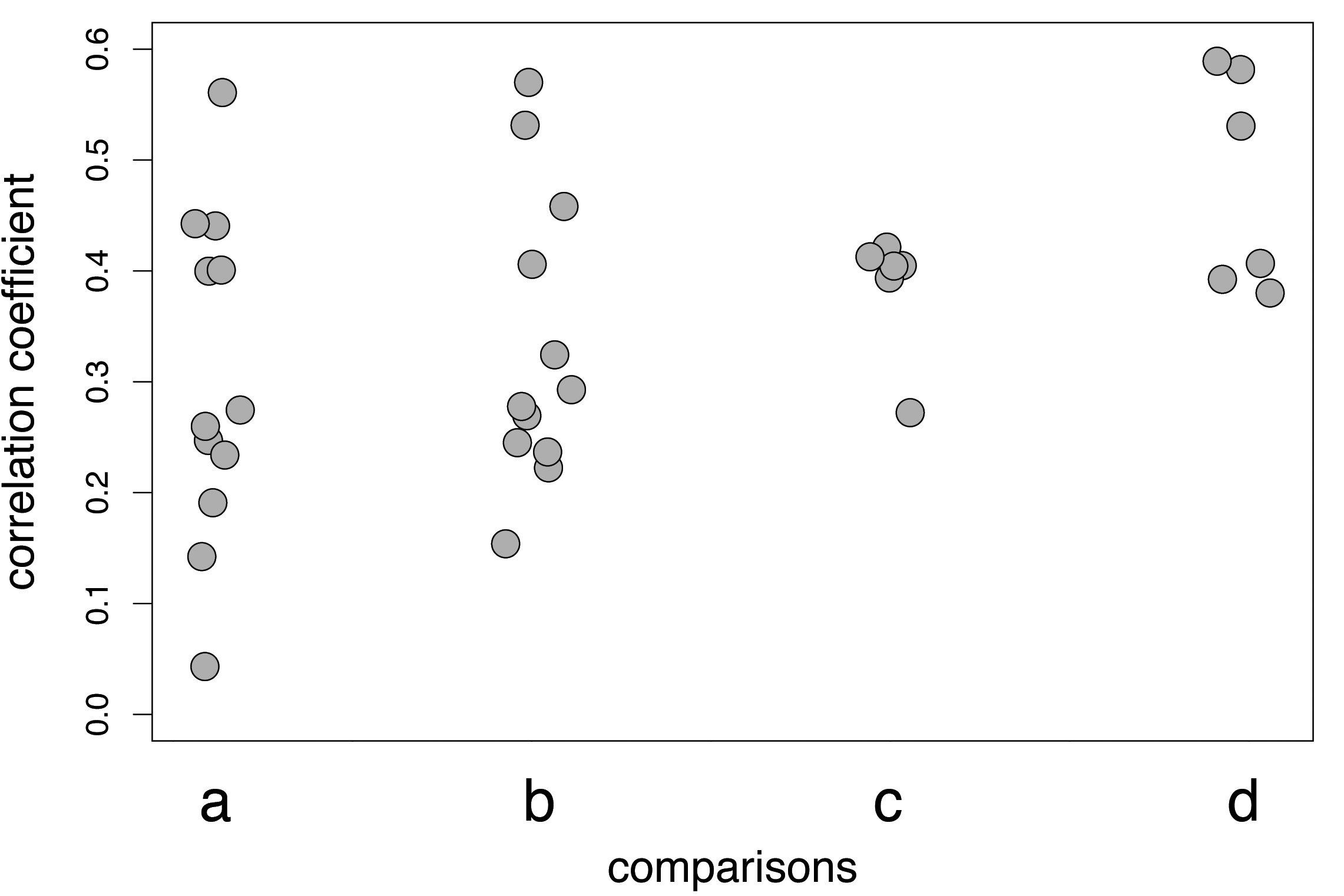


**Figure S5** Distribution of Pearson's correlation coefficients for the comparisons between individuals within a species and between the experimental replicates.

Shown are the correlation coefficients for all 12 pairwise comparisons within the species measured in the two sets of experimental replicates (**a-b**) and 6 comparisons between the pairs of experimental replicates (**c-d**). In **b** and **d**, the points with the Cook’s distance greater than 0.1 were removed prior to the correlation calculation.
